# Comment on: Global variation in anastomosis and end colostomy formation following left-sided colorectal resection

**DOI:** 10.1093/bjsopen/zrab045

**Published:** 2021-05-22

**Authors:** D Schaps, C E Alvarez

**Affiliations:** 1 School of Medicine, Duke University, Durham, North Carolina, USA; 2 Ostomy Service, Saint Vincent of Paul Clinic of the Order of Malta, Santa Tecla, La Libertad, El Salvador

## Abstract

Is the corresponding author a member of a Strategic Partner Society (50% discount on article publication charge):

*Dear Editor*

The article by the GlobalSurg Collaborative^1^ presents data on global disparities in end stoma rates, and concludes that the rate in countries with a low United Nations Human Development Index (HDI) is twice that of middle-HDI countries and three times that of high-HDI countries. We are writing to further the conversation around global stoma disparities by proposing a new indicator for study—stoma quality—based on our experience in a free ostomy-care clinic in Santa Tecla, El Salvador.

El Salvador is considered a medium-HDI country (124 of 189) and, if the article’s conclusions are extrapolated, has higher rates of stoma formation than high-HDI countries. However, even with this presumed higher rate, we have noticed a glaring issue among quality improvement data collected from our population between 2016 and 2020. Namely, the quality of the patients’ stoma creation is poor. Of 419 patients, many have had the stoma created on the flank, sitting over or near bone, not passing through the rectus abdominis muscle sheath, and/or flat, prolapsing, or sunken. This is a disservice to our patients as they have told us anecdotally that the issues with their stomas lead to a significant reduction in quality of life.

It is for this reason that we are calling for a continuation of the GlobalSurg Collaborative’s work by proposing that the global surgical community focuses on the likely disparity in stoma quality affecting low- and middle-HDI nations. More work must be done to evaluate whether this is an issue that affects all low- and middle-HDI nations. If our suspicions are confirmed, global standards for stoma quality must be generated along with programmes to implement the necessary changes. This is a little discussed area of study that, once understood, will allow us to move toward more equitable colorectal surgical care for our patients.

*Disclosure.* The authors declare no conflict of interest.
